# Radiation Attenuation Assessment of Serpentinite Rocks from a Geological Perspective

**DOI:** 10.3390/toxics10110697

**Published:** 2022-11-17

**Authors:** Mostafa A. Masoud, Ahmed M. El-Khayatt, Mohamed G. Shahien, Bottros R. Bakhit, Ibrahim I. Suliman, Ahmed M. Zayed

**Affiliations:** 1 Applied Mineralogy and Water Research Lab (AMWRL), Geology Department, Faculty of Science, Beni-Suef University, Beni Suef 62521, Egypt; 2 Department of Physics, College of Science, Imam Mohammad Ibn Saud Islamic University (IMSIU), Riyadh 11642, Saudi Arabia; 3 Geology Department, Faculty of Science, Beni-Suef University, Beni Suef 62521, Egypt

**Keywords:** serpentinite rock, geochemical and mineralogical composition, morphology, fast neutron, γ-ray, shielding

## Abstract

Serpentinites are metamorphic rocks that are widely applied as aggregates in the production of radiation-shielding concrete. Different varieties of massive serpentinite mountains located in Egypt exist without real investment. Hence, this study aims to evaluate the radiation shielding efficacy of three varieties of serpentinite rocks from different geological perspectives: mineralogical, geochemical, and morphological characteristics. X-ray diffraction, transmitted-light microscopy, and thermal analysis were required to characterize their mineralogical composition, while X-ray fluorescence was necessary to investigate their geochemical features. Moreover, scanning electron microscopy was used to detect their morphological characteristics. On the other hand, the PuBe source and stilbene detector were employed for the experimental determination of fast neutrons and γ-ray attenuations, which were conducted at energy ranges of 0.8–11 and 0.4–8.3 MeV, respectively. Based on the mineralogical, geochemical, and morphological characteristics of these rocks, the radiation attenuation capacity of lizardite > antigorite > chrysotile. However, these serpentinites can be applied as a natural alternative to some radiation-shielding concrete in radiotherapy centers and other counterpart facilities.

## 1. Introduction

Recently, all countries are trying to gather the three equation sides: cost, power supply, and preservation of the environment, to fill the economic gap in energy technologies. Nuclear power can achieve this complex equation. In addition to the high energy density supplied by nuclear reactors, these reactors are not costly in the long term. In addition, the trivial emissions of CO_2_ or other greenhouse gases from these reactors compared to other energy sources enforce our planet protection for future generations [1]. Despite these potential outcomes of nuclear energy, it poses an inevitable threat to humanity. These reactors are vulnerable to radiation leakage or explosion anytime, resulting in more disastrous radiation emissions. On the other hand, the management of these reactor-induced nuclear wastes is another challenge faced by any country that seeks nuclear technology [2]. The threat of these radiations, especially neutrons and γ-rays, lies in their high energy and capability to penetrate the human body [3]. This can lead to more diseases related to cancers and tumors [4]. All previous threats explain the reluctance to use nuclear techniques in practice. Therefore, it was imperative to find a radiation shield for protection from these radiations. As a result, scientists of nuclear engineering, physics, and geology have always sought to find and improve new materials that can effectively shield these radiations in recent years.

Three factors govern the shielding performance of any material. The first is the material density, which is responsible for the attenuation effectiveness against γ-rays as can be illustrated in heavy-weight minerals [5], rocks [6], and heavy-weight concrete [7,8]. The second is the high content of structural or crystalline H_2_O, which is responsible for the attenuation effectiveness of fast neutrons, as demonstrated in hydrous minerals (i.e., high-crystalline H_2_O content) [9] and concrete [10]. The third is suitable additives such as boron or carbon for thermal and fast neutron attenuation, respectively, and this can be observed in minerals of colemanite or boron-containing concrete mixes [11,12,13,14]. Recently, geologists have started to shed light on the effect of the mineralogical composition of minerals and rocks on their radiation shielding performance [10,15]. This trend stems from the principles of applied mineralogy. Applied mineralogy is a branch of geology concerned with the natural barriers comprised of minerals or rocks, which are used to confine radioactive elements [16]. Although concrete is widely applied in natural or artificial crises [13,17], the utilization of raw materials (i.e., minerals and rocks in radiation shielding) has become a method attracting the attention of many studies as an alternative to concrete and cement pastes [18]. This can be credited to many advantages, as follows: (1) lesser cement consumption in concrete and subsequent lesser energy, cost, and CO_2_ emissions, (2) utilization of unexploited mineral resources, (3) space conservation when using thinner shielding walls, (4) less maintenance and longer life compared to concrete. Besides concrete, the lead element is one of the conventional choices to reduce radiation exposure from X-rays and γ-rays. However, it is not encouraged for use because of its toxicity [19]. More specifically, during this period of global economic crisis, local natural rocks are preferred to be used in research to identify the best quality products that can compete in profitably with those currently imported from other countries [20,21]. Moreover, some rocks exhibited superior shielding efficiency over concrete [22,23]. Generally, there are three types of rocks: igneous, metamorphic, and sedimentary. At first, many studies have considered the attenuation properties of igneous rocks, which are categorized into plutonic (subsurface) and volcanic (on the surface) rocks based on the position of formation. Radiation shielding properties of plutonic igneous rocks have attracted the attention of many researchers. Such rocks include dunite, carbonatite [24], peridotite, pyroxenite, gabbro [25,26], syenite [24], granodiorite [18,27], and granite [18,22,27,28,29,30,31,32]. On the other hand, further studies discussed the radiation shielding properties of volcanic igneous rocks such as basalt [6,22,30,33], andesite [18], dolerite [22], rhyolite [6,33], and volcanic tuff [34,35]. Moreover, other volcanic igneous rocks were evaluated by the Monte Carlo method via the Geant4 simulation toolkit for neutrons and the SRIM program for charged particles [36]. The findings proved that one of these rocks was superior to its counterparts in thermal and fast neutrons shielding, as well as charged particles (e.g., electron, alpha, proton, and carbon ion) [37]. As for sedimentary rocks, both clastics (e.g., sandstone) [22] and non-clastics (e.g., limestone) [6,30,33,38] were evaluated as a radiation shield. As for metamorphic rocks such as marble [5,29,30,39,40], metagabbro [26], gneiss, and charnokite [38] were found to be more effective shields against γ-rays compared to concrete [30].

Serpentinite is a metamorphic rock mainly composed of serpentine minerals and associated with minor occurrences of magnetite and carbonate (e.g., dolomite) minerals with a density and crystalline H_2_O ranging from 2.5–2.7 g/cm^3^ and 11–16%, respectively [41]. The differences in magnetite content are one of the main reasons for the variation of serpentinite colour (e.g., grey, greyish black, and green) [42]. Commonly, the most prevalent application of serpentinite rocks is ornamental stones due to their aesthetic characteristics, which renders them commercially named “green marble” [20,43]. A massive series of serpentinite mountains is located in Egypt as a part of the ophiolitic assemblages [44], which are not exploited as ornamental stones due to problems related to their structural and durability properties [45]. Other studies investigated serpentinite rocks as aggregates in normal concrete [46]. Moreover, the serpentine mineral (i.e., serpentinite rock-forming mineral) was investigated as ore, in its native state, for only γ-ray shielding [9] and others considered it as aggregates in the radiation-shielding concrete, RSC, for fast neutron and γ-ray attenuation [47,48,49]. Therefore, all varieties of serpentinite rocks display a common feature that enables them to be eligible for use in radiation shielding. However, to the best of our knowledge, no studies deeply investigated the employment of serpentinite rocks (i.e., in their native status) with their different variations in mineralogy and geochemistry in radiation shielding. This can be understandably attributed to the difficulty of obtaining these rock types separately. Additionally, such rocks are not known by many physicists and engineers. Hence, this study aims to utilize some varieties of Egyptian serpentinite rocks instead of concrete as a geologic repository for nuclear waste disposal or in tile production for lining the walls of radiotherapy centers or nuclear facilities. In addition, the impact of their geochemical, morphological, and mineralogical compositions upon their attenuation ability against γ-rays and fast neutrons using a collimated PuBe source and a stilbene detector will be correlated.

## 2. Materials and Methods

### 2.1. Materials

**Figure 1:**
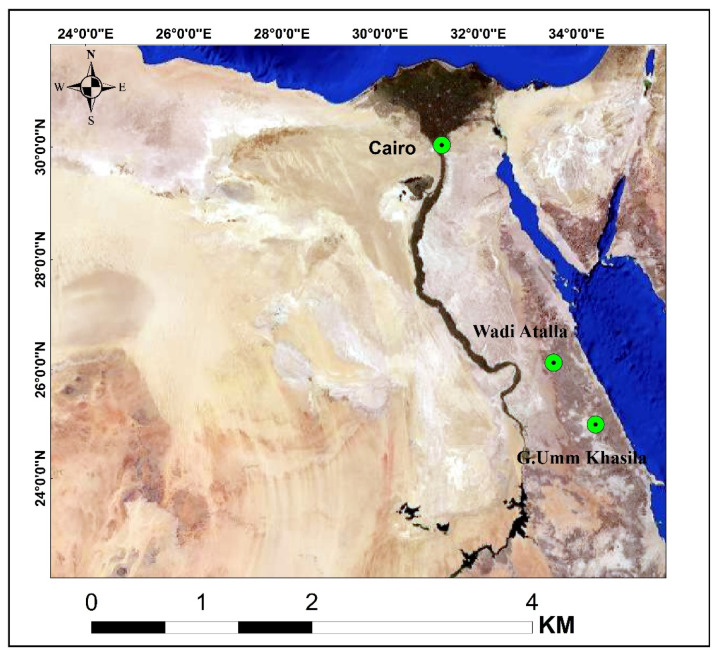
Satellite image showing the locations of the collected serpentinite rocks: AS sample from G. Umm Khasila, as well as LS and CS samples from Wadi Atalla.

Three varieties of serpentinite rocks were sampled from two locations in the Egyptian Eastern Desert, as shown in Figure 1. The antigorite serpentinite sample (AS) was brought from Gabal Umm Khasila, Atud area, along the Marsa Alam-Idfu Road, while lizardite and chrysotile serpentinite samples (LS and CS samples, respectively) were obtained from Wadi Atalla along the Quseir-Qift road in the Red Sea Governorate.

The rock samples were washed to dispose of undesirable materials and then dried. Using a rock cutting saw, three slab samples were sawed out of each rock sample with thicknesses of 2, 4, 6, 8, 10, and 12 cm. As much as possible, the prepared blocks were flattened and leveled to ensure the absence of any gaps when the blocks were positioned together.

### 2.2. Material Characterization

#### 2.2.1. Megascopic Characterization (in the Field)

**Figure 2:**
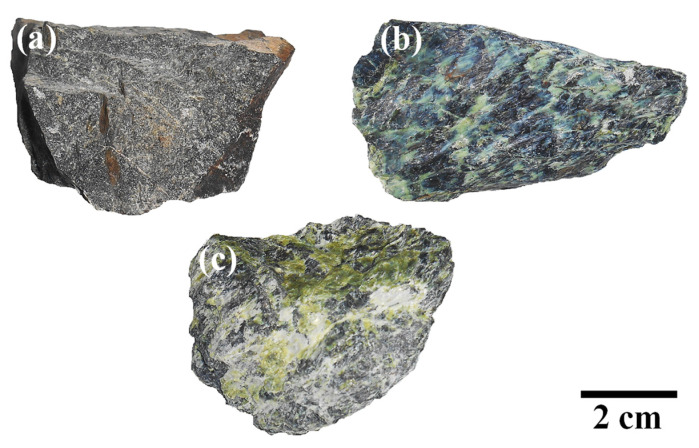
Hand specimens of the three collected samples: (**a**) AS, (**b**) LS and (**c**) CS.

By the visual examination supported by a hand lens, the samples were detected at first sight by their colours, physical properties or weathering. More explicitly, the AS sample is hard with a greenish-black colour, platy grains, and a rough feel (Figure 2a). The LS sample has a greenish blue colour, platy habit, and slippery feel, and is less durable than AS (Figure 2b), while the CS sample contains very fragile and smooth fibres with creamy white colour mottled by pale green (Figure 2c). Moreover, splintery fractures are more prevalent in the LS and especially CS samples.

#### 2.2.2. Mineralogical Composition

Petrographic analyses conducted by transmitted-light microscope (TLM, Nikon, eclipse, LV100POL, Tokyo, Japan) were supported by X-ray diffraction (XRD) to confirm the mineralogical composition of samples. Based on BS EN 12407:2007 [50], thin sections of samples were prepared and examined by the TLM in two optical positions: plane and crossed-polarized light (PPl, and CPL, respectively). Moreover, the Philips X-ray diffractometer (XRD, Mod. PW 139) was equipped with Ni-filtered Cu-Kα radiation to investigate the powdered samples (<63 µm).

#### 2.2.3. Morphological Characterization

Scanning electron microscopy (SEM, JSM-6700F, JEOL Ltd., Tokyo, Japan, with beam energy: 20–30 kV.) was conducted on powdered rock samples (<63 µm) to identify their morphology.

#### 2.2.4. Physical Characterization

These rock samples were designed in specific sizes or shapes, so they were considered dimension stones [51]. The physical properties of the investigated rock samples in terms of density and water absorption are the most substantial and distinguishing characteristics of their specific gravity and porosity, respectively. Thus, the density and water absorption were evaluated based on ASTM C97 [52].

#### 2.2.5. Geochemical Characterization

Complying with the standards of ASTM E1621 and D7348 [53,54], the geochemical analysis was conducted using XRF on the powdered samples (˂63 µm).

#### 2.2.6. Thermal Analysis (TG/DTG)

Thermogravimetry and derivative thermogravimetric (TG/DTG) analyses were applied to determine the relative concentration of crystalline H_2_O (i.e., structural H_2_O) and carbonate content (dolomite, Dol) based on the loss in weight due to their decomposition. Moreover, the TG/DTG analyses are a successful technique to confirm and distinguish between the different phases of serpentine polymorphs [55]. At a heating rate of 10 °C/min, TG/DTG analyses were conducted using SDT Q600 V20.9 Build 20 (TA Instruments, New Castle, DE, USA) under a nitrogen atmosphere at a temperature range of ambient temperature to 1000 °C.

### 2.3. Radiation Measurements

**Figure 3:**
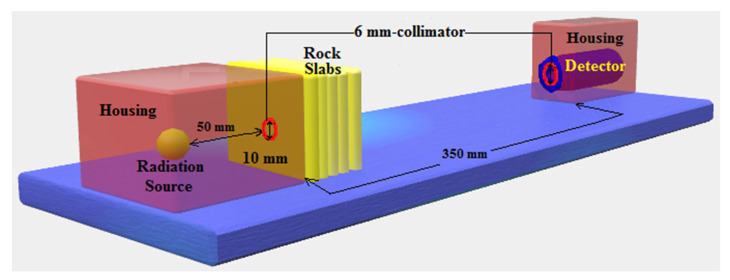
Experimental setup of the conducted radiation measurements.

**Figure 4:**
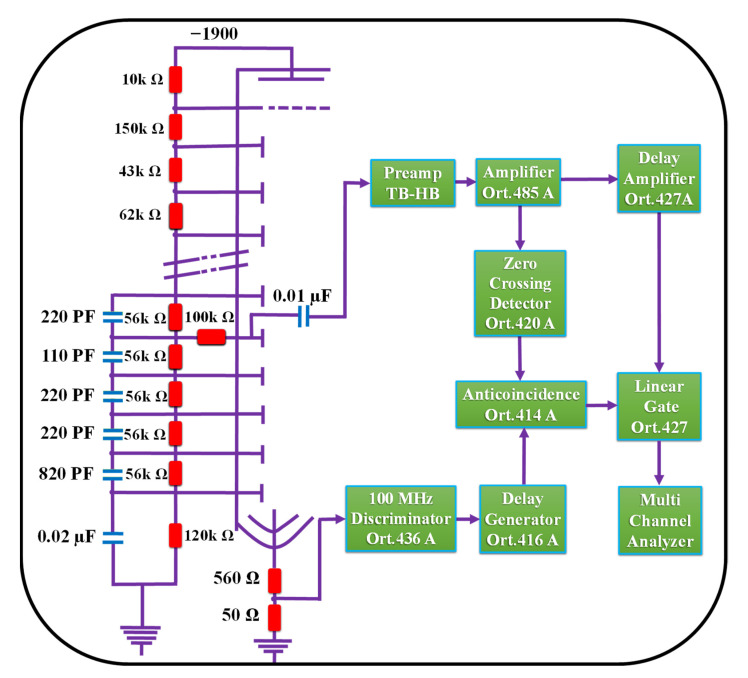
A block diagram of experimental layout showing the electronic device of a fast neutron–gamma spectrometer with dynode assemblages of the photomultiplier tube.

The radiation measurements were implemented at the Nuclear Research Center, Egyptian Atomic Energy Authority. The geometry of fine beam transmission was applied to conduct the shielding measurements against fast neutrons and γ-rays. A narrow-collimated beam emitted from a PuBe source (185 GBq) and a stilbene detector (4 × 4 cm) with a 6 mm aperture was employed. As shown in Figure 3, a fixed distance of 40 cm was maintained between the source and detector. In all radiation measurements, the rock slabs were placed 5 cm away from the PuBe source (Figure 3). The slabs were successively assembled to achieve the required tested thicknesses of 2, 4, 6, 8, 10, and 12 cm. All slabs were leveled to avoid any gaps between them. This experimental setup was located in the room center, and the detector was lead-shielded to minimize the background radiation. A stilbene detector is a common type of organic scintillator widely used in radiation measurements with mixed fields of neutrons and γ-rays. This can be attributed to their perfect properties of pulse shape discrimination (PSD), where neutrons and γ-rays in the scintillator produce signals with different shapes. PSD was conducted via the anticoincidence mode with zero cross-over technique [56] to process the unbidden recoil proton and electron pulses when neutrons and γ-rays interact in the scintillator, respectively. Therefore, PSD was essential to distinguishing between neutron and γ-ray interactions in the detector [57]. The stilbene detector was calibrated using γ-ray spectra at 4.43 and 3.92 MeV emitted from PuBe, as well as 0.661 and 1.332 MeV from ^137^Cs and ^60^Co, respectively. The working energy range of the PuBe source was determined to be within 0.8–11 and 0.4–8.3 MeV for fast neutrons and γ-rays, respectively. Moreover, the energy values of less than 0.8 and 0.4 MeV for fast neutrons and γ-rays, respectively, were not considered to avoid any background radiations, which increase the uncertainties [58]. Additionally, the detector efficiency was unreliable at low energy values [59]. The measuring time was 600 s for every rock sample with 100 s per each slab, other than 100 s for the bare sample, to maintain the statistical uncertainty at ±2%. The experiment was outfitted with a digital counter to track the energy instabilities of the radiation source. Figure 4 illustrates the planned layout of the experiment showing the electronic devices of the neutron–gamma spectrometer with dynode assemblages of the photomultiplier tube.

After conducting the radiation experiments, the attenuation efficiency of fast neutrons and γ-rays was determined by measuring some significant parameters according to the Equations (Equations (1)–(4)) listed in Table 1. Moreover, Equations (5) and (6) were employed to assess the uncertainty propagation, which is <10%.

## 3. Results and Discussion

### 3.1. Megascopic Characterization

As shown in Figure 2, the field inspection illustrated that the serpentinite rocks exhibit a wide range of alterations resulting from the discrepancy in the serpentinization degree (i.e., metamorphism grade). This discrepancy is reflected in the obtained colours, which stem from the variation in mineralogy. Hence, the three samples were selected based on their differences in colour and shape.

### 3.2. Mineralogical Characterization

**Figure 5:**
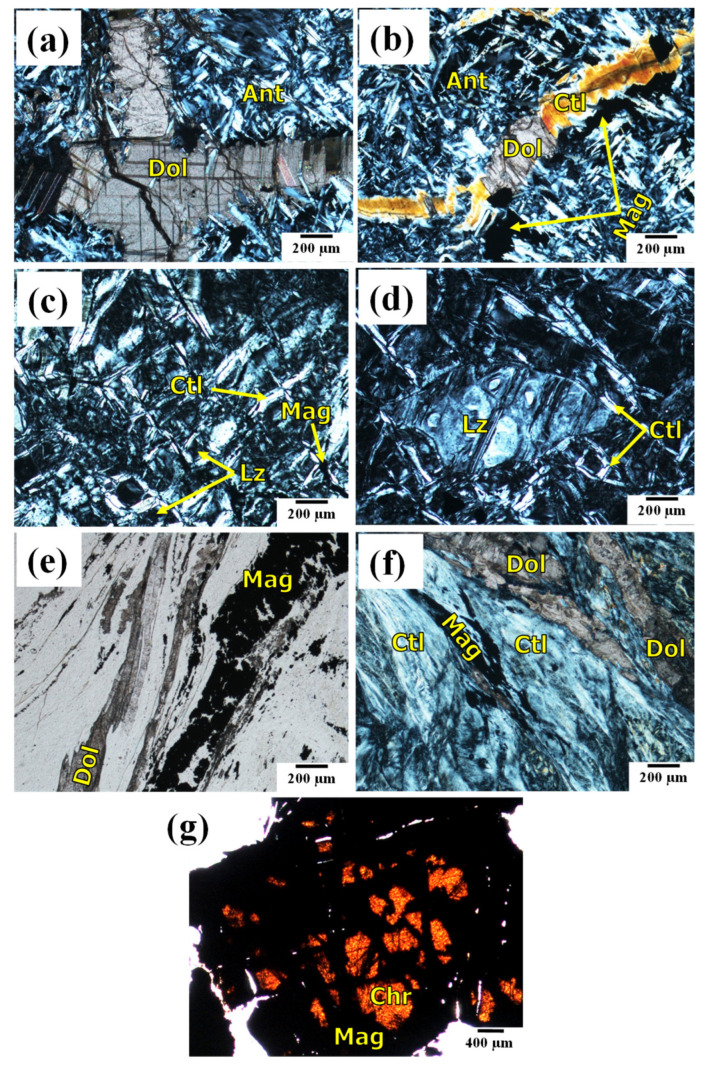
Photomicrographs of serpentinite rocks in PPL and CPL: (**a**,**b**) In AS sample, veinlets of dolomite (Dol), chrysotile (Ctl), and magnetite (Mag) embedded in interpenetrating fibrolamellar antigorite (Ant) in CPL. (**c**,**d**) In LS sample, mesh texture of Lizardite (Lz) surrounded by Ctl and Mag forming hourglass microstructure. (**e**,**f**) In Cs sample, Mag and Dol veinlets crossing groundmass of white Ctl fibres, and (**g**) in CS sample, fresh reddish brown chromite (Chr) in the core corroded with Mag along fractures and peripheries.

The polarizing microscope photomicrographs of the AS sample (Figure 5a,b) illustrate that antigorite (Ant) that occurs as fibrolamellar grains with an interpenetrating texture is the dominant serpentine polymorph with a discernable presence of carbonate minerals (i.e., dolomite, Dol). In addition, a lesser appearance of magnetite (Mag) aggregates is found, while the chrysotile polymorph appears as veinlets cross-cutting the antigorite (Figure 5a,b). Moreover, dolomite can be found in the form of minor inclusions within the chrysotile veinlets due to the carbonation processes of the chrysotile veinlets (Figure 5b). These carbonation processes are one of the chief processes associated with the hydrothermal solutions during serpentinization [60]. The presence of dolomite and chrysotile veinlets filling the shearing microcracks in the AS sample could deteriorate its consolidated structure [61]. As for the LS sample, the lizardite (Lz) serpentine polymorph shows an overwhelming presence with different textures (Figure 5c,d). In Figure 5c, the mesh texture is formed of lizardite surrounded by chrysotile fibres in an hourglass microstructure, while Figure 5d shows a lizardite grain with an augen texture embedded in isotropic grains of lizardite mineral. All previous textures were also reported in earlier studies [62,63]. On the other hand, the existence of chrysotile serpentine polymorph as fibres is prevalent in the CS sample with an observable appearance of sheared lenses and veinlets of dolomite and magnetite minerals (Figure 5e,f). In addition, these aspects can alter the physical properties (i.e., density and water absorption) and the radiation shielding properties as well. Figure 5g illustrates that there are fresh chromite aggregates in CS sample with a reddish brown colour in the core, and magnetite has corroded them at their cracks and edges [64].

**Figure 6:**
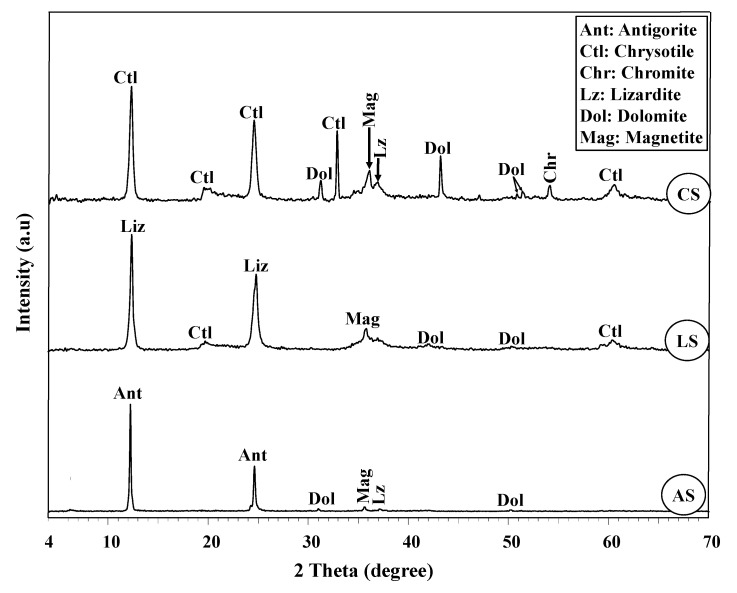
XRD patterns of the three serpentinite rocks, AS, LS, and CS, revealing their mineral composition.

The results of XRD patterns (Figure 6) almost match the findings of polarizing microscopy (Figure 5). The results exhibit that the AS is primarily dominated by highly intense antigorite peaks associated with minor occurrences of lower-intensity peaks of lizardite, dolomite, and magnetite, minerals (Figure 5a,b and Figure 6), while the XRD pattern of the LS sample showed that the lizardite polymorph peaks are the main phase with minor occurrences of chrysotile, magnetite, and dolomite, in that order, following the trend of the polarizing microscope (Figure 5c,d). As for the XRD pattern of the CS sample, chrysotile is a predominant polymorph with minor occurrences of dolomite, magnetite, and chromite, matching with TLM outcomes (Figure 5e–g). Otherwise, the content of lizardite is not high enough in the CS sample to be detected.

### 3.3. Morphological Characterization

**Figure 7:**
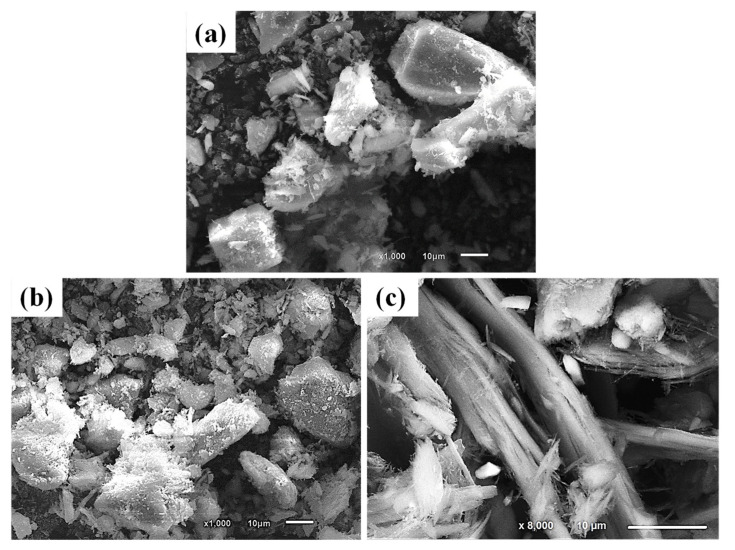
SEM images of the three serpentinite rocks illustrating their surface morphology and shape: (**a**) AS sample has a sub-rectangular shape with a rough surface, (**b**) LS with platy shape and a rough morphology, and (**c**) CS sample contains splintery and fibrous bundles with a smooth surface.

As illustrated in Figure 7, SEM images reveal that the AS sample contains sub-rectangular-shaped particles with euhedral edges and rough surfaces (Figure 7a), while the LS sample contains almost platy shaped particles with rough surfaces as well (Figure 7b). Otherwise, the CS sample resembles splintery bundles with a fibrous habit with smooth surfaces and fissures (Figure 7c).

### 3.4. Physical Characterization

Table 2 illustrates the measured density (g/cm^3^) and water absorption (%), which indirectly characterize the specific gravity and porosity of samples. The variation of density among these samples is inversely proportional to the water absorption variance. More specifically, the AS sample displays the highest value of density (2.60 g/cm^3^), while the CS sample displays the lowest value (2.46 g/cm^3^) with a medium value for the LS sample (2.24 g/cm^3^). These variances in density can be reflected in the consolidation and specific gravity of each sample. On the other hand, contrary to density outcomes, the CS and AS samples display the highest (8.30%) and the lowest (1.58%) water absorption values, respectively, while the LS sample displays a medium one (4.21%). The variation in water absorption can be attributed to the difference in the porosity of the investigated samples in favour of the CS sample, as assured by SEM data.

### 3.5. Geochemical Characterization

Regarding the geochemical composition of the different studied samples, serpentinization is the overarching process responsible for the addressed discrepancies [66]. As shown in Table 3, the chemical composition of the three samples is mainly distributed among MgO, SiO_2_, Fe_2_O_3_, and LOI%. In contrast to MgO%, SiO_2_ and Fe_2_O_3%_ are the highest in the AS and LS samples (40.97, 8.03, and 40.21, 5.88%, respectively), with a preference for the AS sample [67]. This can be credited to the highest ratio of antigorite in AS (Figure 5 and Figure 6), compared to lizardite and chrysotile in the LS and CS samples, respectively. Relating to LOI%, it is inversely proportional to Fe_2_O_3_ and SiO_2_%. More specifically, the AS sample displays the lowest LOI% (11.94%) content, while the CS sample displays the highest LOI% (17.16%) [67] content, and lizardite displays a medium ratio (12.71%). Besides crystalline H_2_O, the carbonate (CO_3_) ratio mainly contributes to the recorded LOI% in the investigated samples. This is more apparent in the LOI% of CS sample than that of LS, and AS samples in descending order. Therefore, the highest LOI% in CS sample can be ascribed to the intensive presence of dolomite that accompanied the high degree of experienced serpentinization [68,69] (Figure 5 and Figure 6).

On the other hand, Al_2_O_3_, CaO, and Cr_2_O_3_% represent the minor components in the addressed samples (Table 3). However, the highest Al_2_O_3_ ratio in the LS sample (1.49%) compared to others can be attributed to the predominance of lizardite polymorph, while CaO% is the highest in the CS sample (3.62%) compared to the AS sample (0.23%) and the LS sample (0.60%) due to a higher ratio of carbonate (i.e., dolomite), as shown in Figure 5 and Figure 6. The minor occurrence of Cr_2_O_3%_, which is the highest in the AS and LS samples (0.34% and 0.36%, respectively), is due to the existence of chromite mineral, as confirmed in Figure 5g. Compared to the AS and CS samples, the higher ratios of Al_2_O_3_ and Cr_2_O_3_ in the LS sample are compatible with previous studies [70].

### 3.6. Thermal Analyses (TG/DTG)

**Figure 8:**
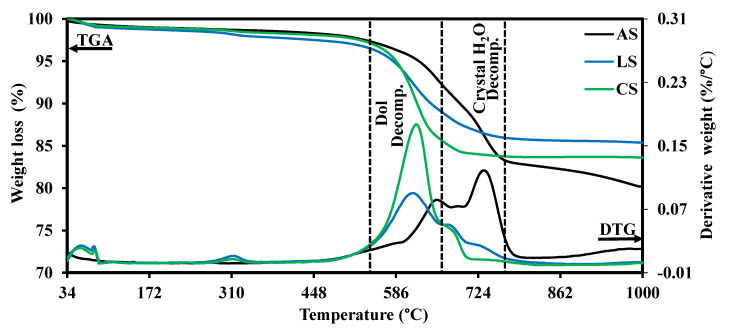
TG/DTG curves of studied rock samples.

TG/DTG curves illustrate that dolomite and crystalline H_2_O are thermally decomposed at 560–680 and 680–785 °C, respectively (Figure 8). These temperature ranges comply with previous studies [55,71,72]. The crystalline H_2_O decomposition implies serpentine mineral dehydroxylation. As depicted in Figure 8, the trend of dolomite decomposition in the investigated serpentinites is inversely proportional to the crystalline H_2_O decomposition. More specifically, the CS sample displays the highest dolomite decomposition, followed by the LS and AS samples. This was confirmed by the findings of TLM and XRD (Figure 5 and Figure 6), which indicate that the CS sample contains the highest dolomite content. On the other hand, the decomposition of crystalline H_2_O displays the highest value for the AS sample, followed by the LS and CS samples, respectively. This corrects the misconception that the LOI% signifies the crystalline H_2_O only without considering the carbonate content (e.g., dolomite, magnesite or calcite), which deceptively increases the LOI%. Therefore, the CS sample contains the lowest crystalline H_2_O content despite containing its highest LOI% (Table 3).

### 3.7. Radiation Measurements

#### 3.7.1. Fast Neutron Attenuation

**Figure 9:**
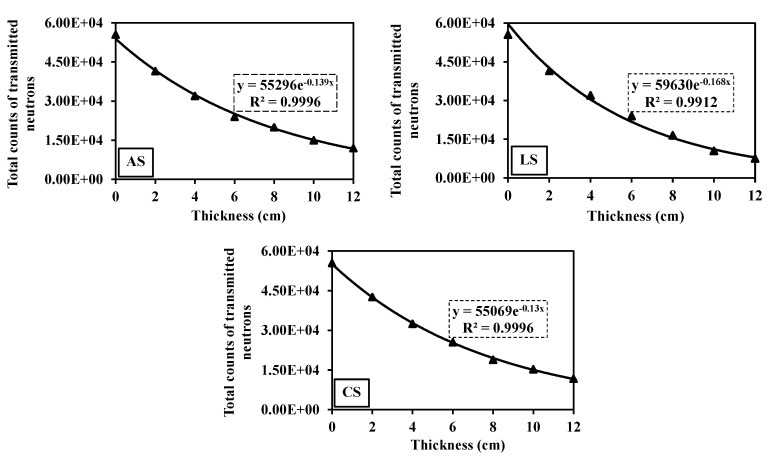
Fast neutrons transmitted behind different thicknesses of the three serpentinite rocks at an energy range of 0.8–11 MeV.

Figure 9 shows that the fast neutron flux is exponentially attenuated with the increase in the rock thickness. This is consistent with the law stated by Beer–Lambert, as illustrated in Table 1. From these exponential relationships, the effective macroscopic removal cross-section of fast neutrons (Σ_R_, cm^–1^) for each sample is obtained. As shown in Table 4, the values of Σ_R_ (cm^–1^), MFP, and HVL (cm) are listed. These attenuation parameters demonstrate that the attenuation of rock samples can be ordered as follows: LS > AS > CS. Although the CS sample contains the highest LOI%, its attenuation ability is the worst compared to others, whereas the LOI% is supposed to increase the hydrogen or the content of the light element leading to a higher likelihood of neutrons to be slowed by scattering processes [6]. However, the high LOI value of the CS sample may be illusionist and does not include the crystalline H_2_O (i.e., structural H_2_O) as expected in most cases [10] in contrast to others such as limonite or goethite [73]. These findings can be readily illustrated by the TG/DTG of rock samples (Figure 8). TG/DTG analyses show that the carbonate (i.e., dolomite, Dol) contributes significantly to the high LOI% of the CS sample. Hence, the LOI is mistakenly estimated as high crystalline H_2_O, and this case is contrary to that in the AS and LS samples, where the significant share of the LOI% is crystalline H_2_O. This deduction is supported by the polarizing microscopy (Figure 5) and XRD analysis (Figure 6). On the other hand, the lower attenuation performance of AS compared to the LS sample can be attributed to the high content of secondary chrysotile and dolomite veinlets filling the microcracks in the AS sample compared to that in the LS sample (Figure 5a–d). These veinlets invalidated the effect of high hydrogen content in AS enough to be reason for its fast neutron attenuation deterioration. More specifically, these veinlets are weak points due to being premium leaking channels for the fast neutron fluxes. Additionally, these veinlets are of lower density, compressibility, and crystalline H_2_O compared to the primary replaced mineral (i.e., antigorite).

#### 3.7.2. γ-Ray Attenuation

**Figure 10:**
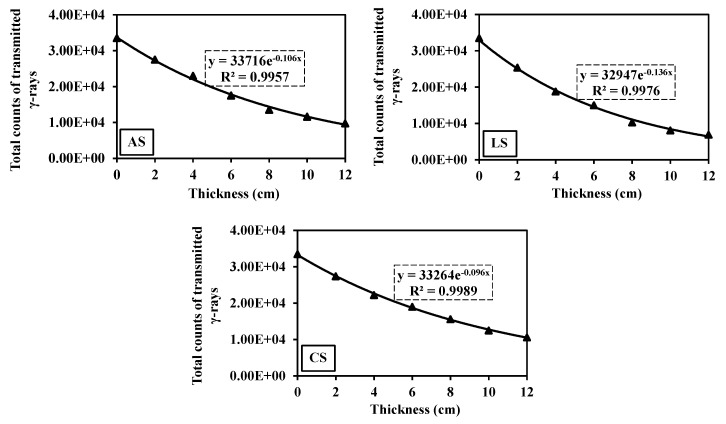
γ-rays transmitted behind different thicknesses of the three serpentinite rocks at an energy range of 0.4–8.3 MeV.

Figure 10 illustrates that the γ-ray flux is exponentially attenuated with the increase in the rock thickness in consistency with the Beer–Lambert law, as shown in Table 1. From these exponential relationships, the linear attenuation coefficient of γ-rays (µ, cm^−1^) for each sample is attained. As shown in Table 5, the values of µ (cm^−1^), MFP, and HVL (cm) are listed. Like the fast neutron attenuation, these γ-ray attenuation parameters demonstrated that the γ-ray attenuation of serpentinite rocks has followed the same tendency, LS > AS > CS (Table 5 and Figure 10). Although the AS sample is of a higher density and Fe_2_O_3%_ (2.6 g/cm^3^ and 8.03%) than the LS sample (2.46 g/cm^3^ and 5.88%, respectively), the γ-ray attenuation of the LS sample is greater (Table 3). This can be imputed to the same reason as in the case of fast neutron attenuation, where the predominance of chrysotile and dolomite veinlets in AS contributes to the worsening of γ-ray attenuation (Figure 5a,b). The high Fe_2_O_3_ content in AS samples also contributed to higher secondary γ-ray emissions via (n, γ) reactions, causing attenuation decline as reported by [47]. On the other hand, the lower performance of the CS sample can be attributed to lower density (2.24 g/cm^3^) and the higher distribution of dolomite and chrysotile veinlets (Figure 5e,f).

Moreover, the attenuation capabilities of the addressed samples against fast neutrons and γ-rays were compared to previous studies, as illustrated in Table 6. Knowing that the comparison was based on the same conditions of radiation measurements, the source type and measurement geometry to obtain a fair comparison. The comparison reveals that the radiation attenuation efficiencies of the addressed rocks are more superior to those of some concrete mixes.

## 4. Conclusions

In this study, the radiation shielding capabilities of three varieties of serpentinite rocks were investigated based on their geological features, and the main findings can be compiled as follows:

1. Inspected serpentinite rocks (AS, LS, and CS) revealed variations in the mineralogical, geochemical, and morphological properties. More specifically, the AS sample is mainly composed of antigorite mineral with high SiO_2_ and Fe_2_O_3%_, while lizardite and chrysotile are the principal minerals of the LS and CS samples, respectively, with lower SiO_2_ and Fe_2_O_3%_. Magnetite and dolomite are the dominant associating minerals in these samples but in different magnitudes. As for the morphological features, AS sample had a sub-rectangular shape with a rough surface, while LS sample had a platy shape with rough morphology, and the CS sample possessed splintery and fibrous bundles with a smooth surface.
2. It was found that the physical, mineralogical, geochemical, and morphological properties of investigated rocks had significant implications for their radiation-shielding behaviour. This was evident in the deleterious impacts prompted by these properties on radiation shielding as follows: (a) in the CS sample, the higher water absorption and lower density, which are indicative of high porosity and lower specific gravity of this sample compared to the AS and LS samples (physical properties), (b) in the AS and CS samples, the higher proportion of lower density dolomite and chrysotile minerals, which are presented as veins in both CS and AS samples (mineralogy), (c) the higher LOI% of the Cs sample works as a false indication for crystalline H_2_O (geochemistry), (d) moreover, in the CS sample, the lower compactness of splintery and fibrous bundles with a smooth surface compared to the higher compactness of sub-rectangular and platy habits with rough surfaces in the AS and CS samples, respectively (morphology).
3. It was found that the radiation shielding behaviour followed the following order: LS > AS > CS, against both fast neutrons and γ-rays. This was correlated with the measured radiation attenuation parameters of fast neutrons and γ-rays, involving µ (cm^−1^), Σ (cm^−1^), MFP (cm), and HVL (cm).
4. Specifically, the radiation attenuation investigation of serpentinite rocks should be taken with caution. This can be assigned to the commonly associated dolomite mineral, which renders the LOI% misleading and illusive ratio for the crystalline H_2_O responsible for fast neutron attenuation. Therefore, LOI% cannot be directly indicative of the amount of crystalline H_2_O compared to other samples such as limonite and goethite.
5. The AS and LS samples are more convenient and competent for radiation shielding compared to the CS sample.
6. The serpentinite rocks are promising rocks as shields against fast neutrons and γ-rays in nuclear facilities considering their mineralogy and geochemistry.
7. With the same radiation measurement conditions (i.e., source type and geometry), the serpentinite rocks are more efficient as a radiation shield than some concrete mixes.

## Figures and Tables

**Table 1:** Equations applied to measure the different attenuation parameters of fast neutrons and γ-rays, as well as uncertainty propagation equations.

No.	Parameter	Symbol	Unit	Description	Equation	Abbreviations
(1)	Effective Macroscopic removal cross-section of fast neutrons	Σ_R_	cm^–1^	probability of fast neutron to undergo a first collision removing it from the group of penetrating, uncollided neutrons	$N=N_{o}e^{-\Sigma_{R}x}$	N_0_ and N are incident and transmitted intensities for fast neutrons, respectively, within the energy range of 0.8–11 MeV. *x:* sample thickness in cm.
(2)	Linear attenuation coefficient of γ-rays	μ	cm^–1^	fraction of attenuated incident photons per unit thickness of a material	$I=I_{0}e^{-\mu x}$	I_0_ and I are incident and transmitted intensities for total γ-ray, respectively, within the energy range of 0.4–8.3 MeV.
(3)	Mean free path	MFP	cm	Average distance between the two successive interactions.	MFP = 1/Σ, 1/Σ_R_, 1/μ	
(4)	Half value layer	HVL	cm	Thickness reducing the radiation intensity to half	HVL= ln 2/Σ, ln 2/Σ_R_, ln 2/μ	
(5)	Uncertainty propagation equations			$\Delta \;(\mu )=\frac{1}{x}\sqrt{{\left(\frac{\Delta \;I_{o}}{I_{o}}\right)}^{2}+{\left(\frac{\Delta \;I}{I}\right)}^{2}+{\left(\ln\frac{I_{o}}{I}\right)}^{2}\left[{\left(\frac{\Delta \;\rho }{\rho }\right)}^{2}+{\left(\frac{\Delta \;x}{x}\right)}^{2}\right]}$		ρ: sample density
(6)				$\Delta (\Sigma_{R})=\frac{1}{x}\sqrt{{\left(\frac{\Delta \;N_{o}}{N_{o}}\right)}^{2}+{\left(\frac{\Delta \;N}{N}\right)}^{2}+{\left(\ln\frac{N_{o}}{N}\right)}^{2}\left[{\left(\frac{\Delta \;\rho }{\rho }\right)}^{2}+{\left(\frac{\Delta \;x}{x}\right)}^{2}\right]}$		

**Table 2:** Physical properties of the studied samples.

Property	AS	LS	CS	International Standard
Density (g/cm^3^)	2.60	2.46	2.24	ASTM C97 and C1526 [52,65]
Water absorption (%)	1.58	4.21	8.30	

**Table 3:** XRF results showing the chemical compositions (%) of studied rock samples.

Oxide (%)	AS	LS	CS
MgO	37.05	38.08	37.39
SiO_2_	40.97	40.21	35.18
Fe_2_O_3_	8.03	5.88	5.69
CaO	0.23	0.60	3.62
Al_2_O_3_	0.80	1.49	0.06
SO_3_	0.02	0.05	0.10
K_2_O	0.04	0.07	0.04
TiO_2_	0.03	0.03	0.01
MnO_2_	0.06	0.08	0.13
P_2_O_5_	0.03	0.00	0.00
Cr_2_O_3_	0.34	0.36	0.11
SrO	0.05	0.00	0.06
NiO	0.36	0.42	0.31
Co_3_O_4_	0.04	0.01	0.12
ZnO	0.00	0.00	0.00
LOI	11.94	12.71	17.16

**Table 4:** Experimental fast neutron attenuation parameters measured behind PuBe with statistical uncertainty calculated from the counting data at (1 σ).

Sample Type	Ʃ_R_ (cm^–1^)	MFP (cm)	HVL (cm)
AS	0.139 ± 0.011	7.19 ± 0.57	4.99 ± 0.40
LS	0.168 ± 0.013	5.95 ± 0.48	4.13 ± 0.45
CS	0.130 ± 0.010	7.69 ± 0.62	5.33 ± 0.43

**Table 5:** Experimental γ-ray attenuation parameters with statistical uncertainty calculated from the counting data at (1 σ).

Sample Type	µ (cm^−1^)	MFP (cm)	HVL (cm)
AS	0.106 ± 0.007	9.43 ± 0.66	6.54 ± 0.46
LS	0.136 ± 0.01	7.35 ± 0.51	5.10 ± 0.36
CS	0.096 ± 0.007	10.42 ± 0.73	7.22 ± 0.51

**Table 6:** Comparison between the radiation attenuation capabilities of addressed rock samples and previously studied concrete mixes.

Sample Code	Description	Σ_R_ (cm^−1^)	µ (cm^−1^)	Ref.
A	Concrete totally composed of antigorite serpentinite aggregate	0.102	0.0832	[13]
AB1	Concrete composed of antigorite serpentinite aggregate incorporated with 1% of boric acid by cement weight	0.105	0.0782	
AB2	Concrete composed of antigorite serpentinite aggregate incorporated with 3% of boric acid by cement weight	0.108	0.0751	
AH25	Concrete composed of 75% antigorite serpentinite aggregate + 25% hematite aggregate	0.1164	0.0880	[47]
AH50	Concrete composed of 50% antigorite serpentinite aggregate + 50% hematite aggregate	0.1402	0.0980	
AB25	Concrete composed of 75% antigorite serpentinite aggregate + 25% barite aggregate	0.1264	0.0910	
AB50	Concrete composed of 50% antigorite serpentinite aggregate + 50% barite aggregate	0.1580	0.1052	
LBC	Concrete totally composed of lizardite serpentinite aggregate	0.0981	0.0785	[10]
CBC	Concrete totally composed of chrysotile serpentinite aggregate	0.0930	0.0761	
AS	Antigorite serpentinite rock	0.139	0.106	Current study
LS	Lizardite serpentinite rock	0.168	0.136	
CS	Chrysotile serpentinite rock	0.130	0.096	

## Data Availability

The authors confirm that the data used to support the findings of this study are available within the article.
